# Clinical characteristics of pediatric allogeneic hematopoietic stem cell transplantation-associated thrombotic microangiopathy (TA-TMA): a retrospective single-center analysis

**DOI:** 10.1007/s12094-023-03129-1

**Published:** 2023-03-28

**Authors:** Linlin Luo, Hao Xiong, Zhi Chen, Li Yang, Ming Sun, Wenjie Lu, Fang Tao, Zhuo Wang, Jianxin Li, Zuofeng Li, Sujie Tang

**Affiliations:** 1 Medical College of Jianghan University, Wuhan, 430056 Hubei Province China; 2 Wuhan Children’s Hospital (Wuhan Maternal and Child Healthcare Hospital), Wuhan, 430016 Hubei Province China; 3 Medical College of Wuhan University of Science and Technology, Wuhan, 430065 Hubei Province China

**Keywords:** Transplant-associated thrombotic microangiopathy, Allogeneic hematopoietic stem cell transplantation, Pediatric, Complement sC5b-9

## Abstract

**Objectives:**

To investigate the clinical features of thrombotic microangiopathy associated with allogeneic hematopoietic stem cell transplantation in children.

**Methods:**

A retrospective analysis of continuous clinical data from HSCT received in the Department of Hematology and Oncology of Wuhan Children's Hospital from August 1, 2016 to December 31, 2021.

**Results:**

During this period, 209 patients received allo-HSCT in our department, 20 (9.6%) of whom developed TA-TMA. TA-TMA was diagnosed at a median of 94 (7–289) days post-HSCT. Eleven (55%) patients had early TA-TMA within 100 days post-HSCT, while the other 9 (45%) patients had TA-TMA thereafter. The most common symptom of TA-TMA was ecchymosis (55%), while the main signs were refractory hypertension (90%) and multi-cavity effusion (35%). Five (25%) patients had central nervous system symptoms (convulsions and lethargy). All 20 patients had progressive thrombocytopenia, with 16 patients receiving transfusion of platelets that was ineffective. Ruptured red blood cells were visible in only two patients with peripheral blood smears. Cyclosporine A or Tacrolimus (CNI) dose was reduced once TA-TMA was diagnosed. Nineteen cases were treated with low-molecular-weight heparin, 17 patients received plasma exchange, and 12 patients were treated with rituximab. TA-TMA-related mortality percentage in this study was 45% (9/20).

**Conclusion:**

Platelet decline and/or ineffective transfusion post-HSCT should be considered an early indicator of TA-TMA in pediatric patients. TA-TMA in pediatric patients may occur without evidence of peripheral blood schistocytes. Aggressive treatment is required once diagnosis is confirmed, but the long-term prognosis is poor.

## Introduction

Allogeneic hematopoietic stem cell transplantation (allo-HSCT) is the most effective curative therapy for several hematological malignancies and bone marrow failure disease in pediatric patients. However, transplantation-related complications provide a serious threat to the long-term survival of patients, one of which is transplantation-associated thrombotic microangiopathy (TA-TMA). TA-TMA is characterized by microvascular hemolytic anemia and thrombocytopenia [1]. The fundamental trigger to onset of TA-TMA is vascular endothelial injury. In addition to the widening of the sub-endothelial space and platelet thrombosis, multiple organ dysfunction also tends to occur particularly of the kidney, heart, intestine, lungs, and brain [2]. Currently, the diagnosis and treatment of TA-TMA in pediatric patients is still in the exploratory stage. In this paper, we retrospectively analyzed the clinical characteristics of 20 pediatric patients with TA-TMA, and hereby present the diagnosis, clinical sequelae, and outcomes.

## Methods

### Study design, patients, and treatment

This study retrospectively analyzed 20 patients who underwent allogeneic HSCT and were confirmed as having TA-TMA in the Department of Hematology and Oncology, Wuhan Children's Hospital from August 2016 to December 2021. TA-TMA was diagnosed according to the criteria proposed by the Hematopoietic Stem Cell Application Group, Chinese Society of Hematology, Chinese Medical Association [3]. The criteria for inclusion were lactate dehydrogenase (LDH) levels increased to upper normal limit, proteinuria, hypertension [4], thrombocytopenia < 50 × 10^9^/L or a 50% decrease in platelet count, decreased hemoglobin, evidence of microangiopathy, and plasma sC5b-9 values above the upper limit of normal (252 ng/mL) [5]. If 3 of the 7 laboratory or clinical indicators were met, or if tissue biopsy indicators had evidence of microthrombosis, then a diagnosis of TA-TMA was confirmed.

Once diagnosis of TA-TMA was established, the dose of cyclosporin and tacrolimus (CNI) was immediately reduced. Plasma exchange, rituximab, low molecular weight heparin, calcium, defibrotide, eculizumab, and other supportive treatments were administered.

### Follow-up endpoints and laboratory metrics

The follow-up endpoint was either June 30, 2022, or patient death. This was achieved by reviewing hospital medical records, laboratory data, conducting telephone interviews, and outpatient reviews. Clinical indicators included clinical manifestations, routine blood tests, platelet transfusion refractoriness (PTR), proteinuria, ruptured red blood cells in peripheral blood, LDH, soluble terminal complement complex sC5b-9, and pathological diagnosis.

### Definition

We define a platelet transfusion–refractory patient as an individual who has received two recent (but not necessarily consecutive) platelet transfusions within a 24 h window, and where corrected count increment (CCI) was lower than 4500/μl [6]. We define positive urine protein as random urine protein exceeding the upper limit of normal value^3^. We use the urine analysis test strip (dry chemical method) of SYSMEX Co., Ltd. to detect urine protein.

### Statistical analysis

Statistical analyses were performed using SPSS, version 24.0 (IBM, Chicago, IL). Continuous variables were expressed as median and range, and the categorical variable was expressed as count with percentage. Hierarchical cluster analysis was used in the clustering.

## Results

### Clinical data

A total of 209 pediatric patients underwent allo-HSCT, of which 20 were diagnosed with TA-TMA. This equates to 9.60% (95% CI = 5.5%–13.6%). The gender split was 11 males (55%) and 9 females (45%), with a median age of 146 (10–190) months. Basic clinical and demographic information for the 20 patients with TA-TMA is shown in Table 1. Primary diseases included aplastic anemia (30%), acute myeloid leukemia (20%), hemophagocytic lymphohistiocytosis (15%) acute lymphoblastic leukemia (5%), Fanconi anemia (5%), myelodysplastic syndrome (5%), mixed-phenotype acute leukemia (5%), mucopolysaccharidosis (5%), and myeloid sarcoma (5%). According to routine clinical practice in our center, haplo-identical donors were a common source of stem cells, with peripheral blood being the main source. The majority of recipients received busulfan (BU)-cyclophosphamide (CY) and anti-thymocyte globulin (ATG) therapy for myeloablative conditioning. Almost all transplant recipients received CNI-based GVHD prophylaxis, and only one case used sirolimus. Nineteen patients (95%) achieved neutrophil and platelet engraftment. The median neutrophil engraftment time was 15 (10–22) days and the median platelet engraftment time was 16 (7–39) days following allo-HSCT. The median count of graft mononuclear cells was 8.335 (0.821–39.6) × 10^8^/kg and the CD34 + cell count was 7.95 (0.353–23.6) × 10^6^/kg.

**Table 1:** Clinical and demographic characteristics of pediatric patients with TA-TMA

No	Gender	Underlying diseases	Age at transplant, months	Graft type	Age at donor, months	Donor-recipient ABO blood group	Conditioning regimen	GVHD prophylaxis	Graft source	Donor-recipient HLA match	MNC counts(X10^8/KG)	CD34 + counts(X10^6/KG)	Neutrophil engraftment time	Platelet engraftment time
1	F	AA	63	HLA-matched sibling	5	O + /O +	Bu/Cy/Flu/ATG	CsA	PBSC	10	8.38	5.17	11	25
2	F	AML	167	Haploidentical	28	O + /O +	Bu/Cy/ATG	PTCy	PBSC	5	17.7	20.05	19	14
3	M	MPS	39	(9/10)HLA-matched unrelated	0	O + /A +	Bu/Cy/Flu/ATG	CsA/MMF	CB	9	0.821	0.353	22	39
4	M	AML	174	(9/10)HLA-matched unrelated	29	O + /B +	Bu/Cy/ATG	CsA/MMF/MTX	PBSC	9	7.4	7.1	12	12
5	M	ALL	72	Haploidentical	27	A + /O +	Bu/Cy/Flu/ATG	PTCy	PBSC	5	26.06	23.6	14	9
6	M	HLH	35	Haploidentical	32	A + /O +	Bu/Cy/Flu/ATG/VP-16	CsA/MMF/MTX	PBSC	7	7.97	4.64	19	37
7	F	MDS	159	Haploidentical	6	B + /B +	Bu/Cy/Flu/ATG	PTCy	PBSC	5	27.04	14.27	16	11
8	M	HLH	153	Haploidentical	39	B + /O +	Bu/Cy/Flu/ALG	CsA/MMF/MTX	BM + PBSC	8	8.25	7.25	Non-engraftment	Non-engraftment
9	F	AA	50	Haploidentical	33	A + /A +	Bu/Cy/Flu/ATG	CsA/MMF/MTX	PBSC	9	8.29	3.62	15	15
10	F	AA	146	Haploidentical	39	A + /AB +	Bu/Cy/Flu/ATG	CsA/MMF/MTX	PBSC	7	7.5	8.65	12	17
11	F	AA	29	(9/10)HLA-matched unrelated	22	O + /AB +	Bu/Cy/Flu/ATG	CsA/MMF/MTX	PBSC	9	7.4	9.4	15	34
12	M	HLH	163	Haploidentical	5	A + /O +	Bu/Cy/Flu/ATG/VP-16	KF506/MMF/MTX	PBSC	5	7.85	5.8	18	23
13	M	MPAL	105	Haploidentical	42	A + /O +	Bu/Cy/Flu/ATG	PTCy	PBSC	6	31.89	13.52	15	15
14	F	FA	10	(9/10)HLA-matched unrelated	28	B + /0 +	Bu/Cy/Flu/ATG	CsA/MMF	PBSC	9	7.4	6.2	10	7
15	F	AML	182	Haploidentical	38	B/AB	Bu/Cy/Flu/ATG	PTCy	PBSC	5	33.9	13.25	17	15
16	M	AML	145	Haploidentical	34	AB/A	Bu/Cy/Flu/ATG	PTCy	PBSC	5	39.6	14.17	19	22
17	M	AA	190	Haploidentical	41	A + /A +	Bu/Cy/Flu/ATG	CsA/MMF/MTX	PBSC	5	7.98	5.12	15	17
18	M	MS	100	Haploidentical	39	B/AB	TBI/Bu/Cy/Flu/ATG	PTCy	PBSC	6	35.99	17.92	13	11
19	F	AA	170	Haploidentical	19	O + /O +	Bu/Cy/Flu/ATG	CsA/MMF	PBSC	5	8.38	4	16	17
20	M	AML	175	Haploidentical	42	A + /O +	Bu/Cy/Flu/ATG	PTCy	PBSC	6	32.2	14.1	16	16

M, male; F, female;AA, aplastic anemia; ALL, acute lymphoblastic leukemia; AML, acute myeloblastic leukemia; FA, Fanconi anemia; HLH, hemophagocytic lymphohistiocytosis; MDS, myelodysplastic syndrome; MPAL, mixed-phenotype acute leukemia; MPS, mucopolysaccharidosis; MS, myeloid sarcoma; HLA, human leukocyte antigen; Bu, busulfan; Cy, cyclophosphamide; Flu, fludarabine; ATG, anti-thymocyte globulin; ALG, anti-lymphocyte globulin; VP-16, etoposide; TBI, total body irradiation; GVHD, graft-versus-host disease; CsA, cyclosporin A; MMF, mycophenolate mofetil; MTX, methotrexate; FK506, tacrolimus; PTCy, post-transplant cyclophosphamide; MNC, mononuclear cells

### Clinical features of pediatric patients with TA-TMA

TA-TMA was diagnosed at a median of 94 days (7–289) post-HSCT. Eleven (55%) patients had early-onset TA-TMA, which happened within 100 days post-HSCT, while the remaining 9 (45%) patients had late-onset TA-TMA, which happened at > 100 days post-HSCT. Early-onset TA-TMA occurred at a median of 56 (7–94) days, and late-onset TA-TMA occurred at a median of 157 (104–297) days. Nine cases were combined stage III-IV-GVHD, of which 6 were late-onset TA-TMA. The most common symptom of TA-TMA was ecchymosis. Seventeen cases had varying degrees of bleeding, mainly in the skin and mucous membranes, with black-purple petechiae as a typical manifestation, while the gastrointestinal tract and urinary system were also involved. Case "5" showed diffuse alveolar hemorrhage. Seven cases suffered from dyspnea, which was manifested as high-flow oxygen inhalation of 3 L/min and above mask oxygen inhalation, CAPA-assisted ventilation, and even mechanical ventilation with other advanced support, where transcutaneous oxygen saturation could be maintained at approximately 95%. Five cases had central nervous system symptoms, which manifested as convulsions and lethargy. The main clinical signs of TA-TMA were hypertension and effusion. The blood pressure of 18 cases was higher than the higher blood pressure values typically observed in children with hypertension of the same age [4]. Furthermore, seven cases had serous cavity effusion, mainly manifesting as pleural effusion and pericardial effusion (Table 2).

**Table 2:** Clinical features of pediatric patients with TA-TMA

No	TA-TMA diagnosis day post-HSCT	Signs and symptoms	The number of antihypertensive medications	With III-IV GVHD	Hb (g/L)	PLT (10^9/L)	PTR	Proteinuria ( +)	Schistocytes	LDH	sC5b-9	Pathological examinations
1	36	Neurological symptoms, hypertension, hematochezia, dyspnea, pericardial effusion	3	No	87	17	Yes	3 +	+	912	–	No thromboembolic
2	46	Hypertension, dyspnea, DAH	2	No	79	14	Yes	0	−	451	354	No thromboembolic
3	94	Neurological symptoms, hypertension, dyspnea	3	Yes	83	31	Yes	1 +	−	1174	-	-
4	94	Hematuria, edema	1	Yes	60	75	No	3 +	−	367	439	No thromboembolic
5	72	Neurological symptoms, hypertension, mucocutaneous bleeding	2	Yes	80	34	Yes	2 +	−	679	245	Thromboembolic
6	7	Neurological symptoms, hypertension, mucocutaneous bleeding, hematochezia, ascites	3	No	123	32	Yes	2 +	−	1463	–	–
7	157	Hypertension	2	Yes	78	62	No	1 +	−	660	329	Thromboembolic
8	13	Neurological symptoms, hypertension, hematochezia, hematuria, ascites	2	No	79	25	Yes	2 +	−	1516	–	–
9	121	Neurological symptoms, hypertension, mucocutaneous bleeding, dyspnea, pleural effusion	2	Yes	79	17	Yes	+ -	−	761	421	Thromboembolic
10	111	Hypertension, mucocutaneous bleeding	3	No	72	33	Yes	+ -	−	533	407	–
11	43	Hypertension, mucocutaneous bleeding	3	No	76	15	No	0	−	958	648	Thromboembolic
12	83	Hypertension, dyspnea, pleural effusion	2	No	49	13	Yes	2 +	−	4034	410	No thromboembolic
13	209	Hypertension, dyspnea, hematochezia	2	Yes	72	34	Yes	2 +	−	696	-	No thromboembolic
14	178	Hypertension, mucocutaneous bleeding, hematochezia	3	Yes	61	22	Yes	0	−	528	392	No thromboembolic
15	289	Hypertension, hematochezia	2	Yes	65	1	Yes	2 +	−	606	937	Thromboembolic
16	75	Mucocutaneous bleeding	1	No	74	3	Yes	2 +	−	350	416	–
17	56	Hypertension, mucocutaneous bleeding	3	No	90	22	No	1 +	+	734	358	–
18	203	Hypertension, mucocutaneous bleeding, hematochezia, dyspnea, pleural effusion	2	No	41	15	Yes	2 +	−	1878	402.45	Thromboembolic
19	104	Hypertension, mucocutaneous bleeding	5	No	89	47	Yes	1 +	−	837	203	–
20	131	Hypertension, mucocutaneous bleeding	3	Yes	75	28	Yes	0	−	599	651	Thromboembolic

DAH, diffuse alveolar hemorrhage. PTR, platelet transfusion refractoriness. Proteinuria: + -: 15 mg/ml, 1 + : 30 mg/ml, 2 + : 100 mg/ml, 3 + : 300 mg/ml, 4 + : 400 mg/ml

The median platelet counts, hemoglobin, and LDH levels were 21 (1–79) × 10^9^/L, 79 (41–106)g/L, and 715 (350–4034) U/L, respectively. All 20 patients had a progressive decrease in platelets, of which 16 patients received transfusion that was ineffective. Proteinuria was positive in 16 cases and 2 patients showed ruptured red blood cells in peripheral blood smears. Fifteen patients were monitored for sC5b-9, of which 13 patients displayed higher levels than the normal range. Pathological examination was performed in 13 cases, and microthrombosis was evident in the microvascular pathological films of 7 of them .

### Treatment and outcomes

The dose of CNI was reduced once TA-TMA was formally diagnosed. Nineteen patients were treated with low-molecular-weight heparin, 17 patients were treated with plasma exchange, and the median number of plasma exchanges was 4 (3–18). Rituximab was used in 12 patients with a median of 1.5 (1–3) administrations. Two patients were treated with eculizumab and one was treated with defibrotide.

Among the 20 patients, 11 responded well to treatment, with hemoglobin and platelets both increasing, LDH levels and blood pressure decreasing, and no new ecchymosis or petechiae on the skin. Nine patients had a poor response to treatment and unfortunately died (45% mortality from diagnosis). The median time of death was 16 (5–77) days after diagnosis, and the main cause of death was disseminated intravascular coagulation and multi-organ failure. Of the 11 patients who were effectively treated, 4 eventually died of sepsis and acute respiratory failure within 136, 177, 279, and 602 days after diagnosis, respectively. Only seven patients survived to the end of the follow-up period and were reported to be healthy (Table 3).

**Table 3:** Treatment and outcome of pediatric patients with TA-TMA

No	CNI	Treatment of CNI	Episodes of TPE	Episodes of Ritux	Other treatments	Efficacy	Outcome	Died of TMA	Time from diagnosis to death (days)
1	CsA	Withdrawal	5	0	LMWH	Invalid	Died	Yes	14
2	CsA	Reduction	4	0	LMWH	Valid	Survival	–	–
3	CsA	Withdrawal	4	1	LMWH	Invalid	Died	Yes	20
4	CsA	Withdrawal	5	2	LMWH	Valid	Survival	–	–
5	CsA	Withdrawal	4	0	LMWH	Valid	Died	No	–
6	CsA	Reduction	0	0	LMWH	Invalid	Died	Yes	59
7	CsA	Withdrawal	4	1	LMWH	Valid	Died	No	–
8	CsA	Reduction	4	0	Defibrotide	Invalid	Died	Yes	16
9	CsA	Withdrawal	18	3	LMWH/Eculizumab	Valid	Survival	–	–
10	CsA	Withdrawal	14	3	LMWH	Invalid	Died	Yes	77
11	CsA	Withdrawal	3	1	LMWH	Valid	Died	No	–
12	FK506	Withdrawal	3	1	LMWH	Invalid	Died	Yes	7
13	CsA	Withdrawal	13	0	LMWH	invalid	Died	Yes	45
14	CsA	Reduction	4	1	LMWH/Eculizumab	Valid	Died	No	–
15	CsA	Reduction	4	0	LMWH	Invalid	Died	Yes	11
16	CsA	Reduction	4	0	LMWH	Valid	Survival	–	–
17	CsA	Reduction	4	2	LMWH	Valid	Survival	–	–
18	CsA	Withdrawal	0	1	LMWH	Invalid	Died	Yes	5
19	CsA	Reduction	3	3	LMWH	Valid	Survival	–	–
20	CsA	Withdrawal	0	3	LMWH	Valid	Survival	–	–

CNI, calcineurin inhibitor; TPE, therapeutic plasmatic exchange; Ritux, rituximab; LMWH, low-molecular-weight heparin

## Discussion

TA-TMA is a serious complication following hematopoietic stem cell transplantation, with incidence varying widely from 0.5% to 76% [7]. In recent years, as the number of patients with TA-TMA has increased, Epperla [8] concluded that the 1-year incidence in a single center was highest at 12% (95% CI = 9%–15%), while the incidence of the disease in the first year after transplantation in our center was slightly lower at 9.60% (95% CI = 5.5%–13.6%).

The onset time of TMA was approximately 27 days and 303 days after transplantation [9], stratified by early-onset TA-TMA and late-onset TA-TMA, respectively. The time of onset of TA-TMA patients in our center was also distributed in two groups, with a similar number of patients with early-onset and late-onset, and 6 of the 9 late-onset patients (66.6%) had combined grade III–IV°GVHD while 3 of the 11 early-onset patients(27.3%). The differences did not reach statistical significance, potentially due to the limited sample size of this small cohort. For patients receiving long-term, continuous use of cyclosporine and tacrolimus, clinicians need to be alert to the occurrence of late-onset TA-TMA, as these appear to be risk factors for TA-TMA. This conclusion needs to be confirmed by studies with larger sample sizes.

The existing diagnostic criteria [7, 10, 11] have been continuously improved in the last few years. In 2014, sC5b-9 detection was incorporated into the TA-TMA diagnostic criteria [12]. In our study, sC5b-9 was monitored in 15 cases, of which 13 cases had levels exceeding the normal range, while the other two cases were elevated before the diagnosis of TA-TMA. A single sC5b-9 value may not corroborate diagnosis, and iterative testing of sC5b-9 may be instructive in the diagnosis and treatment of TA-TMA.

Schistocytes visible on peripheral blood smears were included in multiple diagnostic criteria. However, one case was reported without peripheral blood schistocytes but with evidence of microthrombosis in pathological examinations of the tissue [13]. It should be noted that expert consensus across China has broader diagnostic criteria [3], with pathological diagnosis or the presence of 5 of the 7 markers being the minimum required for diagnosis. Dvorak [14] believes that elevated LDH, proteinuria, and hypertension may be enough for consideration of TA-TMA diagnosis. In our study, only two patients had peripheral blood schistocytes, which is similar to Young [15] who proposed that peripheral blood schistocytes were not an early predictor of TA-TMA.

Fifteen patients had early clinical manifestations such as platelet transfusion without therapeutic response, skin petechiae and ecchymosis. Platelet monitoring is a routine blood test for pediatric patients within 1 year of transplantation. The appearance of ecchymosis and petechiae is more intuitive than that of proteinuria and hypertension. Unexplained platelet transfusion ineffectiveness and new petechiae may emerge as one of the early diagnostic indicators for the diagnosis of TA-TMA. However, the number of cases in the current study is small, which still needs to be verified by more centers in order to confirm sensitivity and specificity for TA-TMA.

Withdrawal of the calcineurin inhibitors such as cyclosporine and tacrolimus seems to be involved in the treatment of TA-TMA [16], but patients with GVHD need to be evaluated for replacement or reduction of anti-rejection drugs. Plasma exchange is one of the most common methods for the treatment of patients with thrombotic thrombocytopenic purpura at present, but it has poor efficacy in TA-TMA [17]. In recent years, Jodele demonstrated through a retrospective study that early use of plasma exchange may be beneficial for patients with multiple organ failure caused by TA-TMA [18]. Yang [19] has shown that plasma exchange was effective in TA-TMA patients without gastrointestinal bleeding, and it is possible that the frequency and duration of plasma exchange might achieve better efficacy. Plasma exchange was used in 17 patients in this study, where clinical symptoms improved and disease progression slowed down. However, only six patients survived. In particular, the effect of plasma exchange was not good in patients with bleeding.

McFadyen [20] has proposed that endothelial cell damage caused by an immune response to infection could resolve microthrombosis through an anticoagulant pathway. The pathogenesis of TA-TMA is also influenced by the development of microthrombi caused by endothelial injury. Tissue plasminogen activator and low-molecular-weight heparin may be future treatments for TA-TMA. Low-molecular-weight heparin-calcium infusion was used in 19 patients, but its exact efficacy was unclear due to the combination of other treatments. Rituximab is a CD20 monoclonal antibody whose role in TA-TMA is unclear. In the review by Kim [21], 12 of 15 patients (80%) who received rituximab resulted in therapeutic efficacy. In our study, twelve patients were treated with rituximab, of which four experienced ineffective response. However, rituximab is often used in combination with plasma exchange, and the exact effect is not clear in TA-TMA. Defibrotide has been shown to exhibit antithrombotic and fibrinolytic activities. Higham [22] demonstrated that defibrotide may reduce the risk of TA-TMA in pediatric patients. One patient in our center was given defibrotide but it did not work. Eculizumab is a recombinant human monoclonal antibody that inhibits terminal complement C5. The antibody has high affinity for C5, blocks the formation of C5a and C5b-9, and protects vascular endothelial cells from C5b-9-mediated damage. In a study of 64 pediatric patients with high-risk TA-TMA [23], 36 (56%) patients treated with eculizumab achieved complete remission and 5 (8%) achieved a partial response. The remaining 23 (36%) had no response at the end of treatment. Eculizumab improved 1-year post-HSCT survival. Two patients in our center were treated with eculizumab and both achieved remission, but both patients also developed serious infections after its use. Further research is needed for the prevention of treatment complications arising from eculizumab.

In conclusion, TA-TMA is a serious complication after allo-HSCT, with a high mortality rate. Early identification and prompt treatment are key to saving lives. TA-TMA in pediatric patients may occur without evidence of peripheral blood schistocytes. Unexplained precipitous thrombocytopenia or ineffective platelet transfusions may be the earliest signs of TA-TMA in pediatric patients and could be early diagnostic indicators. However, multi-center studies are needed to provide sufficient patient numbers in confirming our observations.

## Data Availability

The data that support the findings of this study are available on request from the corresponding author. The data are not publicly available due to privacy or ethical restrictions.
